# Changes in the free amino acid composition of *Capsicum annuum* (pepper) leaves in response to *Myzus persicae* (green peach aphid) infestation. A comparison with water stress

**DOI:** 10.1371/journal.pone.0198093

**Published:** 2018-06-01

**Authors:** Victoria Florencio-Ortiz, Susana Sellés-Marchart, José Zubcoff-Vallejo, Georg Jander, José L. Casas

**Affiliations:** 1 Instituto Universitario de Investigación CIBIO (Centro Iberoamericano de la Biodiversidad), University of Alicante, Carretera de San Vicente del Raspeig, s/n, San Vicente del Raspeig, Alicante, Spain; 2 Genomics and Proteomics Unit, Servicios Técnicos de Investigación, University of Alicante, Carretera de San Vicente del Raspeig, s/n, San Vicente del Raspeig, Alicante, Spain; 3 Departamento de Ciencias del Mar y Biología Aplicada, University of Alicante, Carretera de San Vicente del Raspeig, s/n, San Vicente del Raspeig, Alicante, Spain; 4 Boyce Thompson Institute for Plant Research, Ithaca, NY, United States of America; Natural Resources Canada, CANADA

## Abstract

Amino acids play a central role in aphid-plant interactions. They are essential components of plant primary metabolism, function as precursors for the synthesis of defense-related specialized metabolites, and are major growth-limiting nutrients for aphids. To quantify changes in the free amino acid content of pepper (*Capsicum annuum* L.) leaves in response to green peach aphid (*Myzus persicae* Sulzer) feeding, plants were infested with a low (20 aphids/plant) or a high (200 aphids/plant) aphid density in time-course experiments ranging from 3 hours to 7 days. A parallel experiment was conducted with pepper plants that had been subjected to water stress. Factor Analysis of Mixed Data revealed a significant interaction of time x density in the free amino acid response of aphid-infested leaves. At low aphid density, *M*. *persicae* did not trigger a strong response in pepper leaves. Conversely, at high density, a large increase in total free amino acid content was observed and specific amino acids peaked at different times post-infestation. Comparing aphid-infested with water-stressed plants, most of the observed differences were quantitative. In particular, proline and hydroxyproline accumulated dramatically in response to water stress, but not in response to aphid infestation. Some additional differences and commonalities between the two stress treatments are discussed.

## 1. Introduction

Although they are best known as constituents of proteins, amino acids also play a central role in a wide variety of other plant physiological processes [1]. They act as osmolytes, regulate ion transport, modulate stomatal opening, participate in detoxification of heavy metals, contribute to redox-homeostasis, influence gene expression, and affect the synthesis and activity of some enzymes [2]. Moreover, amino acids serve as precursors for numerous plant secondary metabolites that fulfill critical functions such as signaling, defense, interactions with other organisms, and photoprotection [1, 3]. Many plant studies have demonstrated accumulation of free amino acids (FAA), especially proline, in response to both abiotic and biotic stresses (reviewed in [1]), including the two explored in the present study: water stress and aphid herbivory.

Aphids are among the most economically important pests in world agriculture. The negative impact of aphids is related in part to their huge reproductive capacity, which leads to high population densities and significant nutrient withdrawal from the plants in the form of phloem sap [4, 5]. Phloem sap is an unbalanced food for aphids, being composed primarily of sucrose and other carbohydrates, as well as nitrogen in the form of FAA [5, 6]. Although amino acids are also present in cells as protein-bound forms with defensive functions [7], aphids are considered to rely primarily on FAA for their nutritional requirements [8]. As phloem-feeders, aphids cannot utilize FAA contained in other leaf cells. Nevertheless, a high correlation in the amino acid composition of whole leaves and phloem exudates has been shown [9], and there is evidence that aphids gain fitness benefits from the total amino acid content of the plant tissue from which they are feeding on [10]. Moreover, phloem changes induced by aphids appear to be systemic, affecting at least the whole attacked leaf [11].

The abundance of essential amino acids (EAA) of phloem sap is too low for animal dietary requirements [5, 6]. To circumvent this problem, aphids contain endosymbiotic bacteria from the genus *Buchnera* that provide them with EAA [6]. Nevertheless, some aphids have been suggested to manipulate plant metabolism to favor their own nutritional requirements, increasing the phloem amino acid content, in particular EAA. This phenomenon of “nutritional enhancement” [11] has been observed in the phloem or bulk leaf tissue of plants after infestation with aphids that cause macroscopic changes in their hosts, including *Schizaphis graminum* [11], *Diuraphis noxia* [11, 12] and *Aulacorthum solani* [13], which produce chlorotic lesions, as well as *Tetraneura* spp. [10] and *Phloemyzus passernii* [7], which produce galls and pseudogalls, respectively. In the case of “asymptomatic” aphids, which do not cause macroscopic changes in their host plants, results have been more variable. Whereas *Aphis glycines* [14] and *Acyrthosiphon pisum* [15] altered FAA composition of their host plants, *Megora viciae* [15] or *Sitobion avenae* [16] did not. Additionally, *Rhopalosiphum padi* feeding did not affect the phloem amino acid content [11], but increased the total FAA content in whole leaves [16].

Due to the dual function of amino acids in plant-aphid interactions, as precursors for the production of many plant defense compounds and as major growth-limiting nutrients for aphids, aphid-infested plants are hypothesized to upregulate FAA biosynthesis, but at the same time limit herbivore access to these nutrients [17]. The large volumes of sap that aphids ingest to acquire sufficient nitrogen can reduce the water potential and induce drought-stress symptoms in plants [18]. The abscisic acid signaling pathway, which is important for drought stress responses in plants, is also induced by aphid feeding [19]. Therefore, it has been suggested that some of the phenotypic changes associated with aphid infestation may be induced by water stress rather than directly by the aphids themselves [20]. The aim of the present work was therefore to investigate changes in the FAA composition of pepper (*Capsicum annuum*) leaves caused by the asymptomatic aphid *Myzus persicae* (Sulzer) and determine whether these changes are similar to those occurring during water stress.

## 2. Materials and methods

### 2.1. Plant material

*Capsicum annuum* var. California Wonder seeds (Ramiro Arnedo S.A, Murcia, Spain) were germinated in plastic pots with a 1:1 mixture of peat (Prohumin potting soil, Projar S.A., Valencia, Spain) and vermiculite. Plants were watered three times each week and maintained in a growth chamber under a 16:8 hr photoperiod (day/night), 24°C, and 70% relative humidity.

### 2.2. Aphid culture and plant infestation

A culture of the green peach aphids (*Myzus persicae* Sulzer) was derived from a population on greenhouse-grown sweet pepper close to Pilar de la Horadada (Alicante), Spain. This stock culture was maintained on pepper plants in a growth chamber under a 16:8 hr photoperiod (day/night), 24°C, and 70% relative humidity.

Pepper plants (five weeks after sowing) were infested sequentially with wingless adult aphids to obtain plants at 3 hours post-infestation (hpi), 8 hpi, 1 day post-infestation (dpi), 2 dpi, 4 dpi and 7 dpi. In order to compare high and low aphid density, plants were infested with 200 or 20 adult aphids, respectively, in two independent assays. In both cases, fifteen plants were assayed at each time point of infestation, and the same number of uninfested plants was used as a control. In each experiment, all leaves were collected at the same time (6 weeks after planting), insects were brushed off and the plant leaves from each treatment were pooled together. Leaves were initially frozen in liquid nitrogen during collection, frozen at −80°C for further freeze-drying, and finally ground and stored at 4°C until analysis.

### 2.3. Water stress

To induce water stress pepper plants were subjected to water constraint. For this, two groups of fifteen plants were maintained under the conditions described above, but without watering for 7 or 14 days, respectively. An additional group of fifteen plants remained watered regularly (3 times/week) and served as controls. The onset of the water constraint treatment was planned sequentially in order to collect all leaves at the same time (6 weeks after planting), and the plant leaves from each treatment were pooled together. As above, leaves were freeze-dried, ground, and stored at 4°C until analysis.

### 2.4. Samples and standards preparation

FAA extraction was performed in quadruplicate from 5 mg of dried leaf tissue in 1 ml of water, with 2 mg.L^-1^ cystine as the internal standard. After homogenization by vortexing, samples were incubated for 10 min at room temperature, centrifuged at 10,000 g for 10 min, and supernatants filtered through a 0.45 μm pore membrane filter (Teknokroma S.A, Spain). Calibration standards were prepared in water by spiking the 21 amino acids analyzed at concentrations from 0.25 to 10 mg.L^-1^. Amino acid standards were purchased from Sigma–Aldrich (St. Louis, MO, USA), with exception of *L*-arginine, which was from Duchefa Biochemie (Haarlem, The Netherlands).

### 2.5. Quantitation of FAA by multiple reaction monitoring

The FAA analysis was carried out by UHPLC-MS/MS using an Agilent 1290 Infinity UHPLC System coupled to an Agilent 6490 triple quadrupole mass spectrometer with an Agilent Jet Stream ion source in positive ionization mode, according to previously published methods [21, 22]. Separation of analytes was performed on an Agilent Zorbax Extend-C18 column (2.1 × 50 mm, 1.8 μm), which was maintained at 25°C during the analysis. In optimized conditions, the mobile phase consisted of solvent A (0.05% formic acid and 0.03% heptafluorobutyric acid (HFBA) in water) and solvent B (0.05% formic acid and 0.03% HFBA in acetonitrile) using the following gradient: 0 min 0% B, 2.5 min 0% B, 5.5 min 40% B, 5.60 min 90% B, 6 min 90% B; at a constant flow rate of 0.4 mL.min^-1^. In order to improve glutamate quantification, a specific chromatographic method was created, consisting of: solvent A (0.5% formic acid and 0.3% HFBA in water) and solvent B (0.5% formic acid and 0.3% HFBA in acetonitrile) using the gradient 0 min 0% B, 2.5 min 0% B, 3 min 40% B, 3.5 min 90% B, 4 min 90% B; at a constant flow rate of 0.4 mL/min. For all the samples, the injection volume was 1 μL.

The multiple reaction monitoring (MRM) analysis mode was used to monitor the transitions from precursor ions to dominant product ions. The optimized source parameters were: gas curtain temperature 275°C, gas flow 11 L min^-1^, cell acceleration voltage 2 V, nebulizer pressure 50 psi, capillary voltage 3000 V and dwell time 10 ms. Several specific transitions were used to determine each compound and, for each transition, the collision energy applied was optimized to detect the greatest possible intensity.

A total of 21 amino acids was analyzed: alanine (Ala), arginine (Arg), asparagine (Asn), aspatate (Asp), cysteine (Cys), glutamine (Gln), glutamate (Glu), glycine (Gly), histidine (His), hydroxyproline (Hyp), isoleucine (Ile), leucine (Leu), lysine (Lys), methionine (Met), phenylalanine (Phe), proline (Pro), serine (Ser), threonine (Thr), tryptophan (Trp), tyrosine (Tyr) and valine (Val). The specific MRM transitions used for quantitation of each amino acid and the optimized MRM parameters, such as fragmentor voltage and collision energy, are summarized in S1 Table.

A MassHunter Workstation (version B.07.01) was used for data acquisition. MassHunter Qualitative Analysis (version B.07.00) and Quantitative Analysis Software (version B.07.00) were used for data processing. The two most abundant MRM transitions were selected for each analyte as quantifier and qualifier ions. Quantification was made according to the internal standard cystine.

### 2.6. Data analysis

Data analysis was conducted with the statistical software R 3.4.0 [23]. The effects of high aphid density, low aphid density and water stress on FAA composition were assessed with Multivariate Analysis of Variance (MANOVA), followed by a post hoc analysis to detect significant differences between times of treatment. Post-hoc tests were conducted with Tukey’s HSD or Games-Howell, depending on whether or not Levene’s test showed homogeneous variance.

In order to compare the changes in FAA under the different treatments, the fold change for each amino acid in the treated relative to the control leaves was calculated by: (bi−ā)/ā, with “bi” being the different replicates of treated plants and “ā” the mean of the corresponding control leaves. In accordance to Dadd [24] we have considered Arg, His, Ile, Leu, Lys, Met, Phe, Thr, Trp and Val as EAA.

To explore whether the global FAA response was different among treatments, we conducted a Factor Analysis of Mixed Data (FAMD). FAMD performs Principal Component Analyses on continuous variables and Multiple Correlation Analyses on categorical variables, enabling the simultaneous analysis of both kinds of factors [25]. The comparison between the treatments was conducted only after 7 days of stress, which was a common duration for all three treatments. Another FAMD analysis between high and low aphid density treatments was conducted to study the possible interactions between aphid density and time of infestation.

## 3. Results

**Fig 1:**
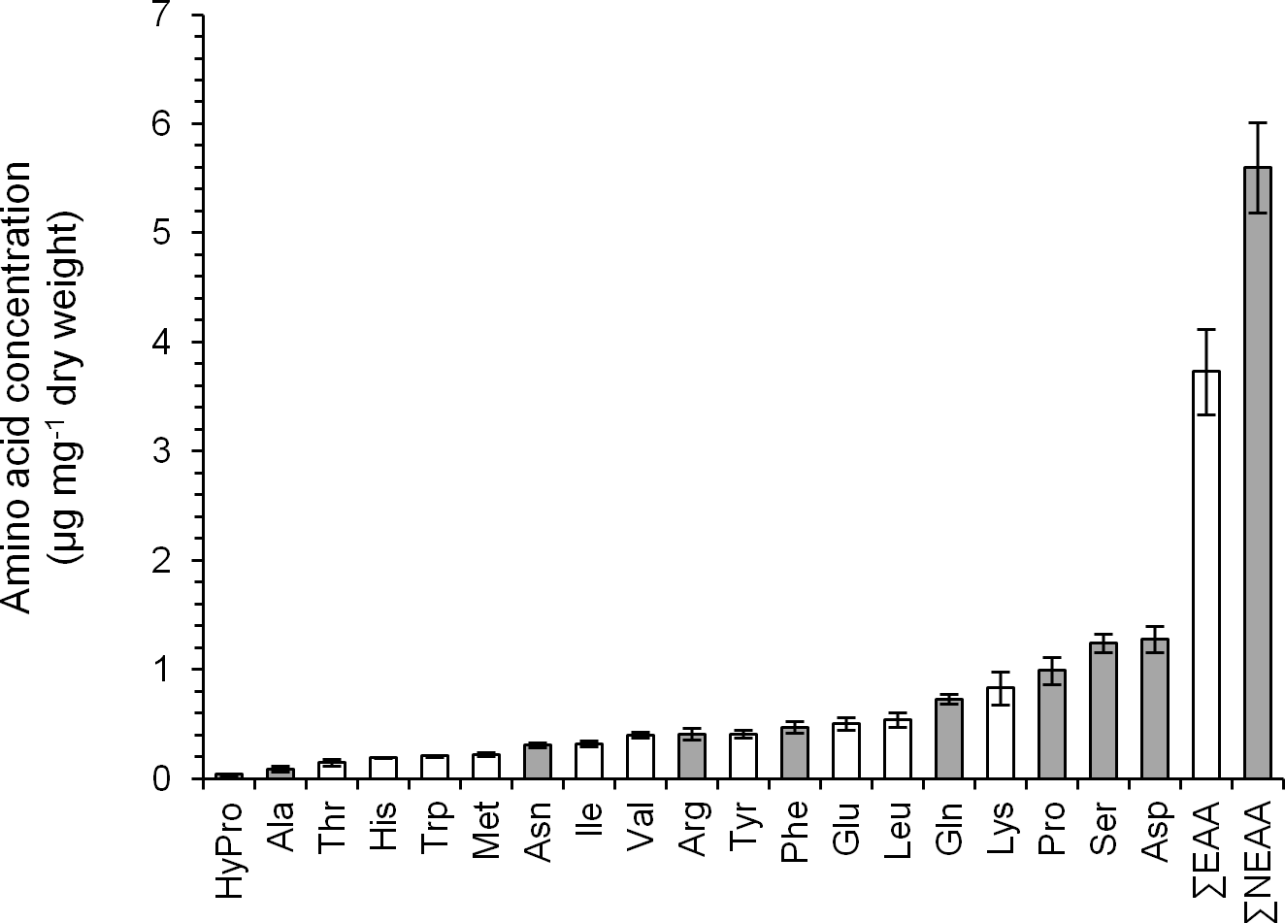
FAA content of control pepper leaves. Mean +/- s.e. of n = 12. White and gray bars correspond to EAA and NEAA, respectively.

FAA analysis of leaves was performed on pepper plants under high aphid density, low aphid density, or water stress, as well as on the corresponding control plants. In all cases, 19 amino acids, but not Cys and Gly, were detected. Fig 1 shows the concentration of each amino acid, as well as EAA and non-essential amino acids (NEAA) content, found in control leaves.

### 3.1. Effect of aphid infestation on plant FAA composition

**Fig 2:**
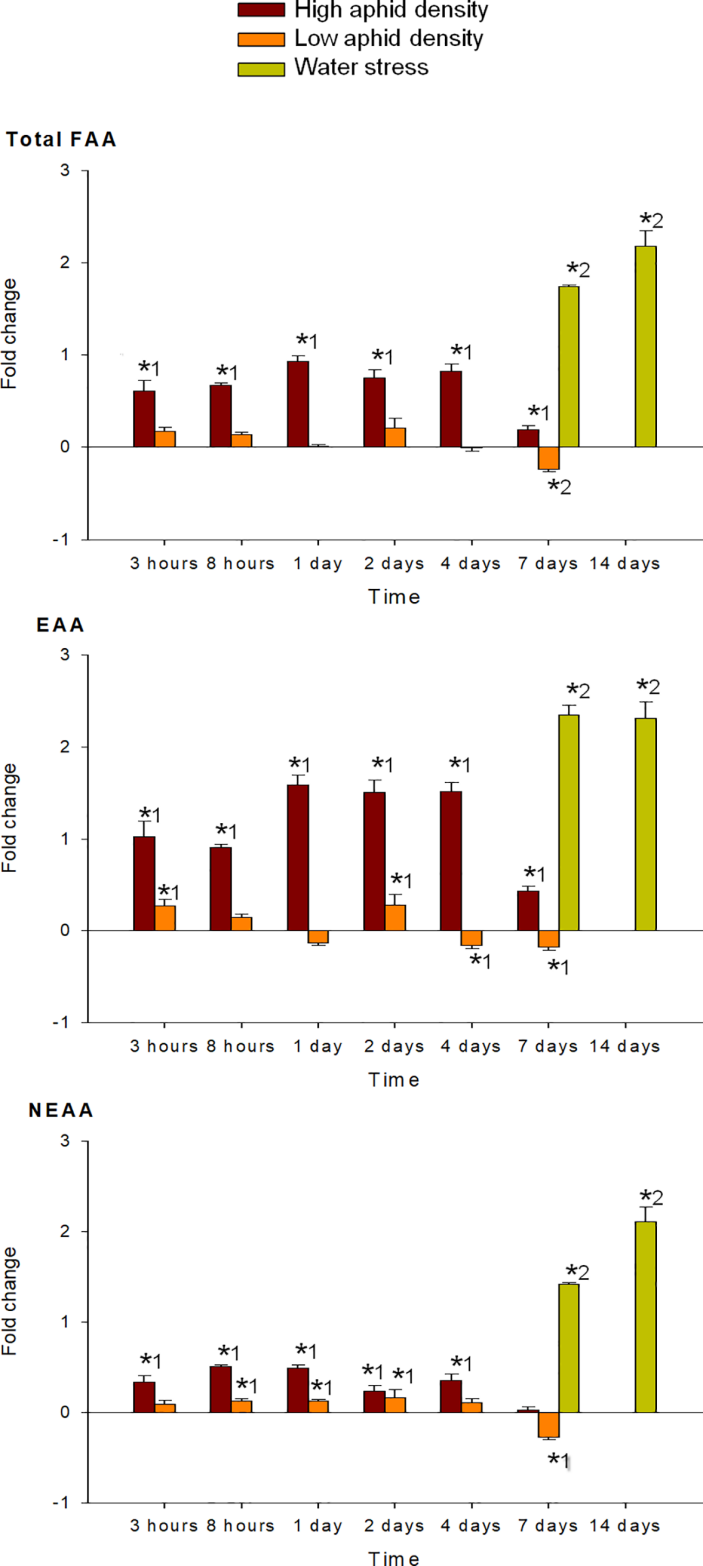
Fold change in total FAA, EAA and NEAA of pepper leaves after different treatments (high aphid density, low aphid density, and water stress). Mean +/- s.d. of n = 4. Positive or negative values indicate increases or decreases, respectively. Asterisks indicate significant changes (P-value < 0.05) in Tukey^1^ or Games-Howell^2^ post hoc analysis relative to their respective control leaves.

Aphid infestation induced significant changes in the total FAA content of pepper leaves at high aphid density, whereas changes of lower magnitude were observed at low aphid density (Fig 2). When plants were subjected to high aphid density, the total FAA fold change was already significant at 3 hpi, increased to maximum levels between 1 dpi and 4 dpi, and decreased thereafter. This increase in the total FAA content was mainly due to an increase in EAA rather than in NEAA. Conversely, at low aphid density the total FAA fold-change was not significant until 7 dpi, when a slight decrease was registered, and the fold changes detected in EAA were very similar to those of the NEAA (Fig 2).

**Fig 3:**
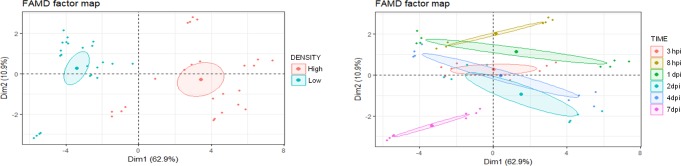
FAMD factor map of FAA in pepper leaves subjected to high- and low-density aphid infestation for different times.

FAMD revealed groups markedly differentiated in their FAA composition, depending on both the density of aphid infestation and the time of infestation (Fig 3). The two first dimensions explained 73.8% of the total variability. Dimension 1 is composed, in order of descending contribution (S2 Table), by the quantitative variables Val, Thr, Phe, Arg, Lys, Ile, Tyr, Leu, Ala, Met, Asn, Trp, and His, and the qualitative variable “density”. On the other hand, dimension 2 is composed, in order of descending contribution (S2 Table), by the qualitative variable “time” and the quantitative variables Glu, Pro, Ser, Gln, and Asp.

Variability between high- and low density aphid treatments is mostly explained by the variables belonging to dimension 1. On the other hand, variability between the different times of infestation is explained similarly by dimensions 1 and 2. Comparing the different times of infestation, 7 dpi is most clearly differentiated from the other time points. Moreover, density x time interactions are pointed out, given that groups based on the time of infestation were not chronologically ordered on dimension 2.

**Fig 4:**
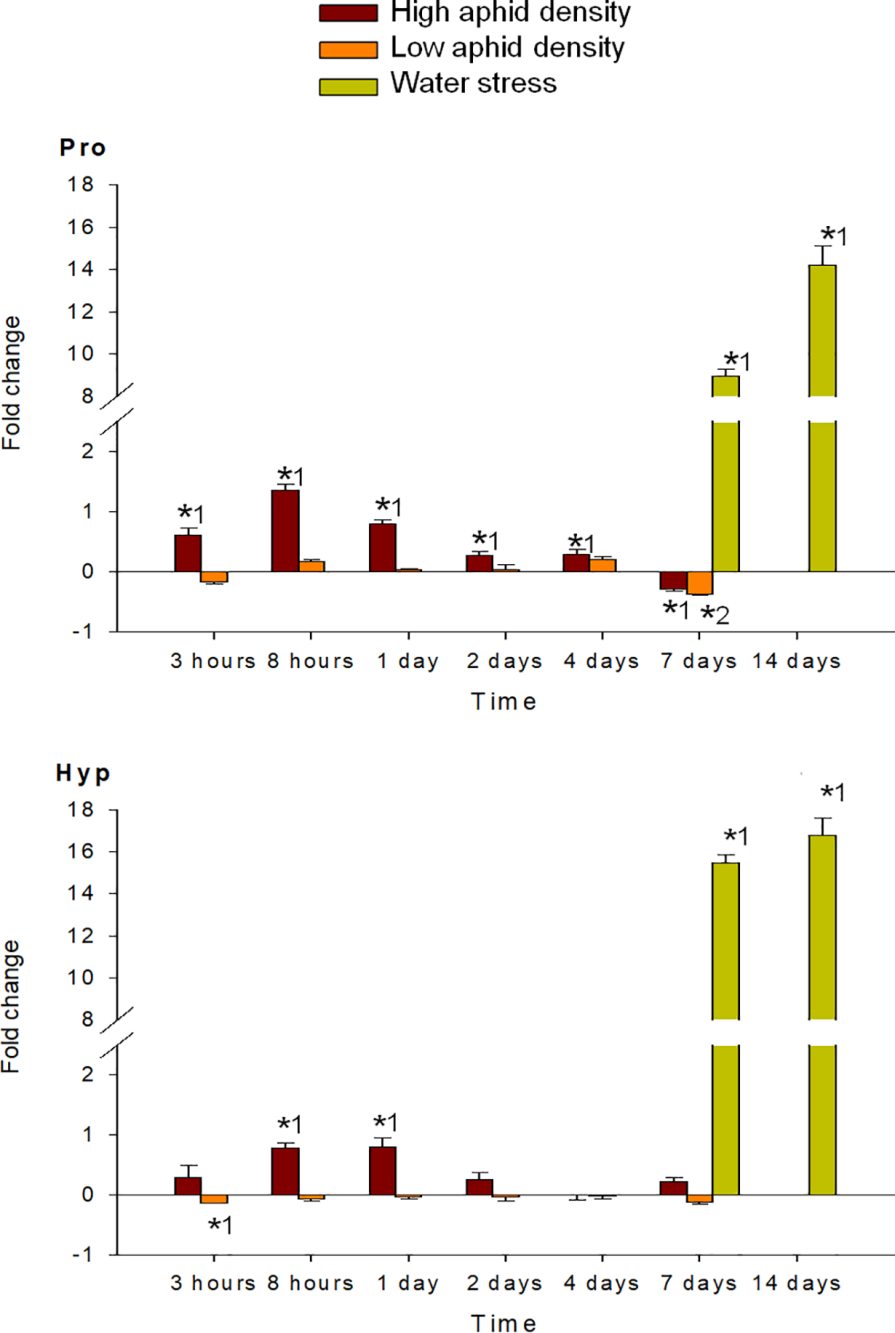
Fold change in Pro and Hyp of pepper leaves after different treatments (high aphid density, low aphid density, and water stress). Mean +/- s.d. of n = 4. Positive or negative values indicate increases or decreases, respectively. Asterisks indicate significant changes (P-value < 0.05) in Tukey^1^ or Games-Howell^2^ post hoc analysis relative to their respective control leaves.

**Fig 5:**
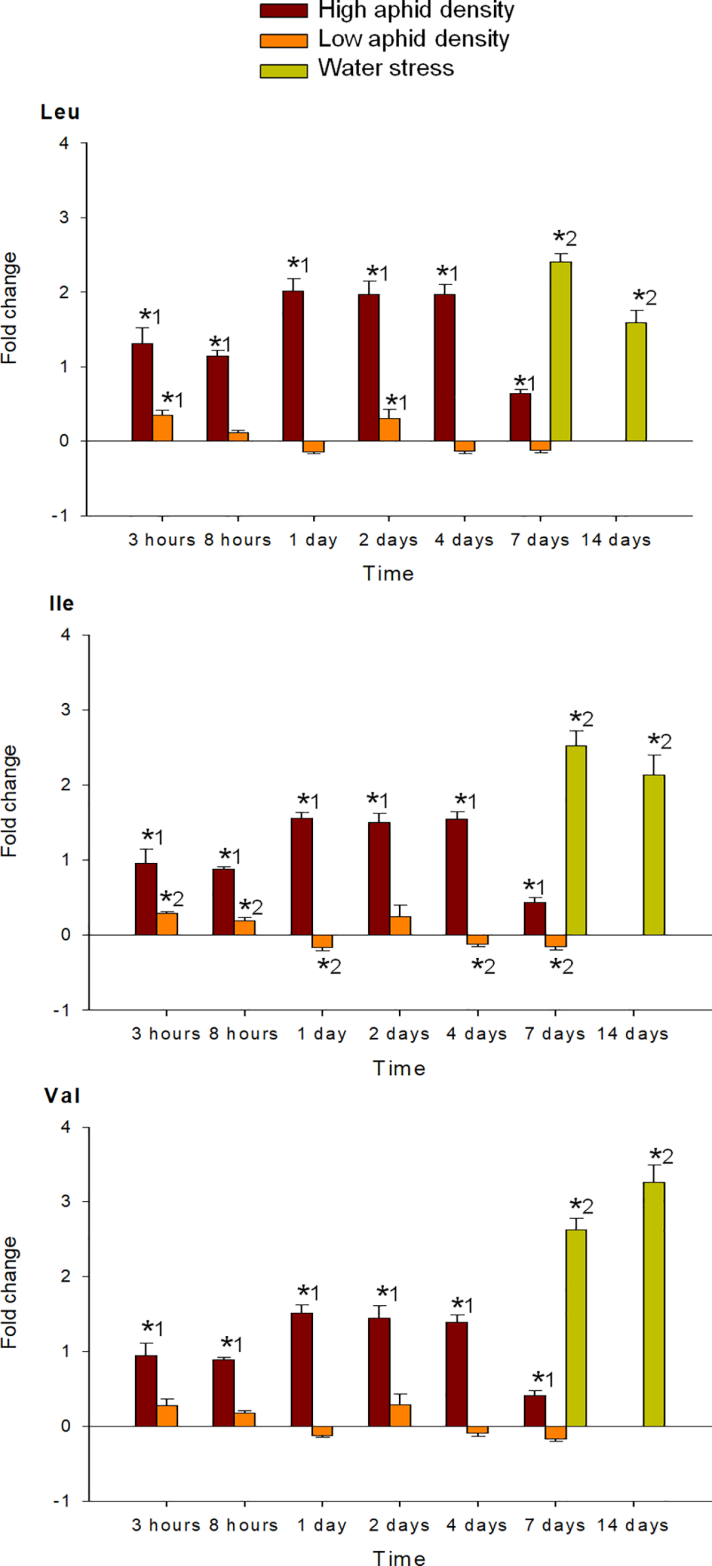
Fold change in Leu, Val and Ile of pepper leaves after different treatments (high aphid density, low aphid density, and water stress). Mean +/- s.d. of n = 4. Positive or negative values indicate increases or decreases, respectively. Asterisks indicate significant changes (P-value < 0.05) in Tukey^1^ or Games-Howell^2^ post hoc analysis relative to their respective control leaves.

**Fig 6:**
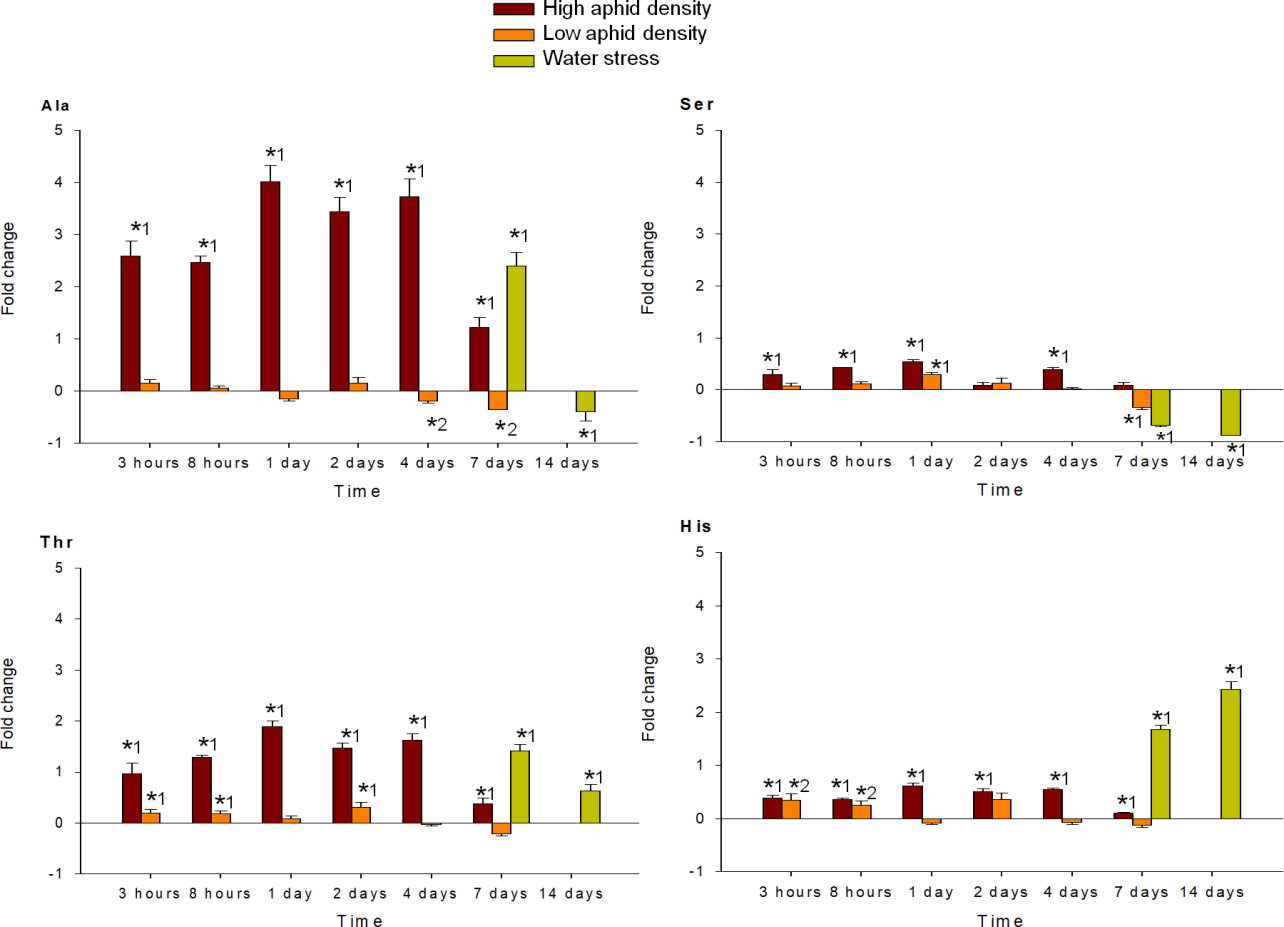
Fold change in Ala, Ser, Thr and His of pepper leaves after different treatments (high aphid density, low aphid density, and water stress). Mean +/- s.d. of n = 4. Positive or negative values indicate increases or decreases, respectively. Asterisks indicate significant changes (P-value < 0.05) in Tukey^1^ or Games-Howell^2^ post hoc analysis relative to their respective control leaves.

**Fig 7:**
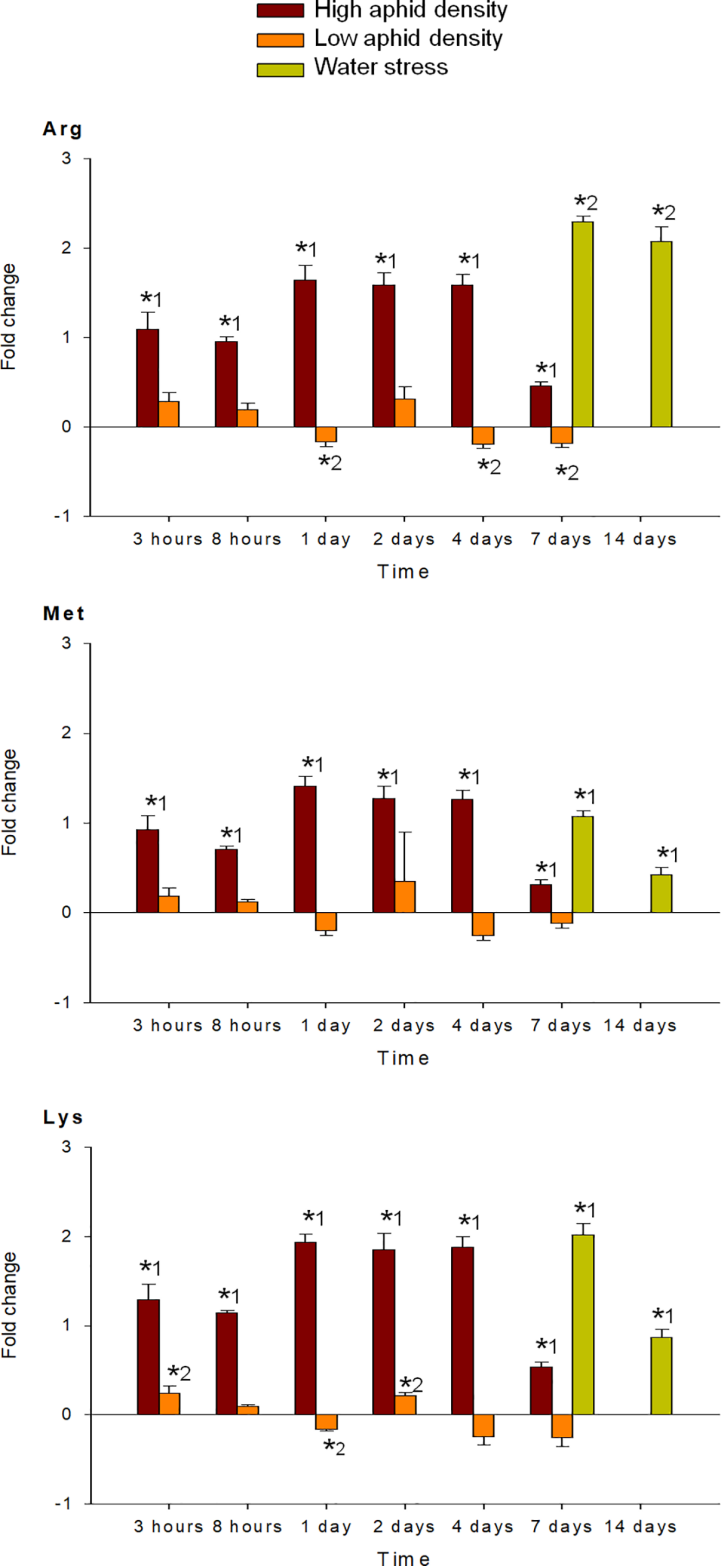
Fold change in Arg, Met and Lys of pepper leaves after different treatments (high aphid density, low aphid density, and water stress). Mean +/- s.d. of n = 4. Positive or negative values indicate increases or decreases, respectively. Asterisks indicate significant changes (P-value < 0.05) in Tukey^1^ or Games-Howell^2^ post hoc analysis relative to their respective control leaves.

**Fig 8:**
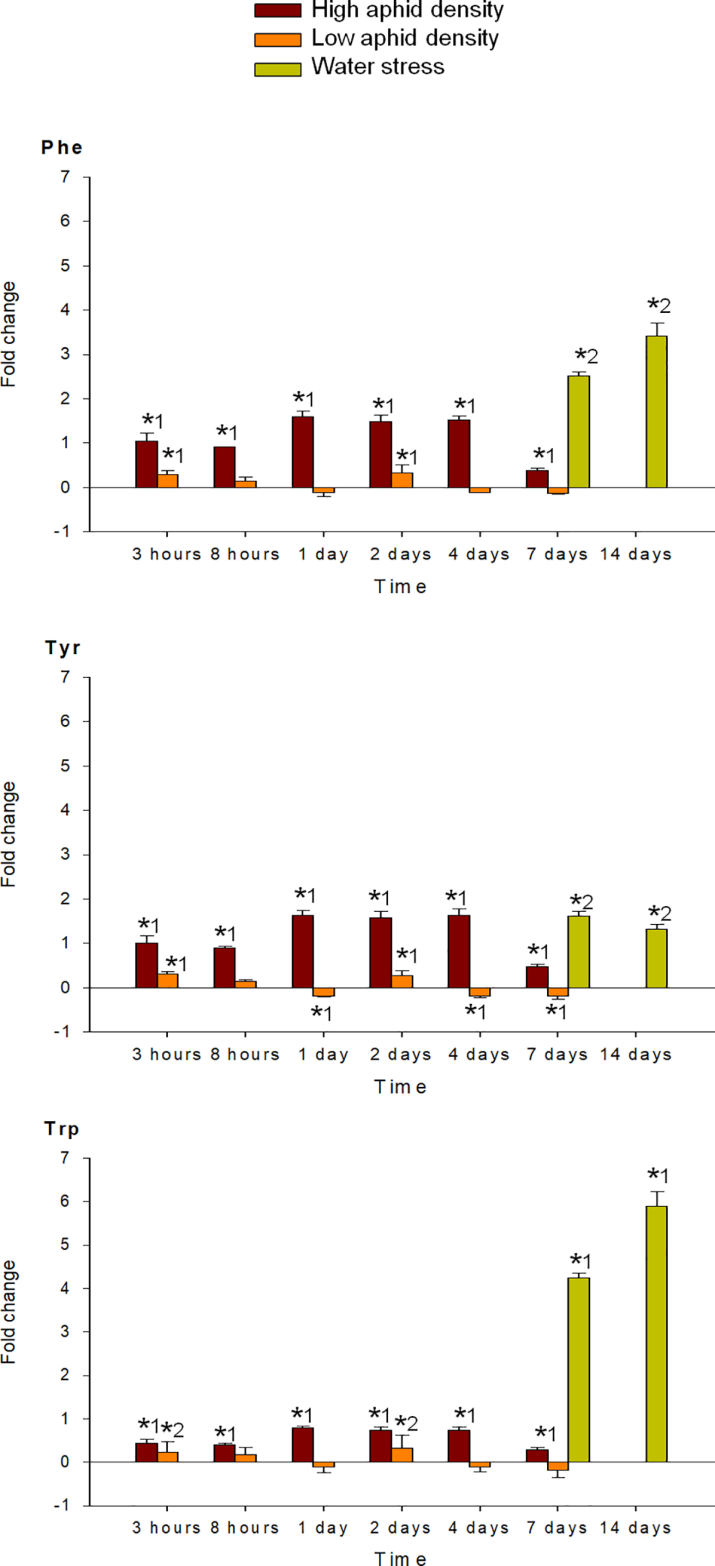
Fold change in Phe, Tyr and Trp of pepper leaves after different treatments (high aphid density, low aphid density, and water stress). Mean +/- s.d. of n = 4. Positive or negative values indicate increases or decreases, respectively. Asterisks indicate significant changes (P-value < 0.05) in Tukey^1^ or Games-Howell^2^ post hoc analysis relative to their respective control leaves.

**Fig 9:**
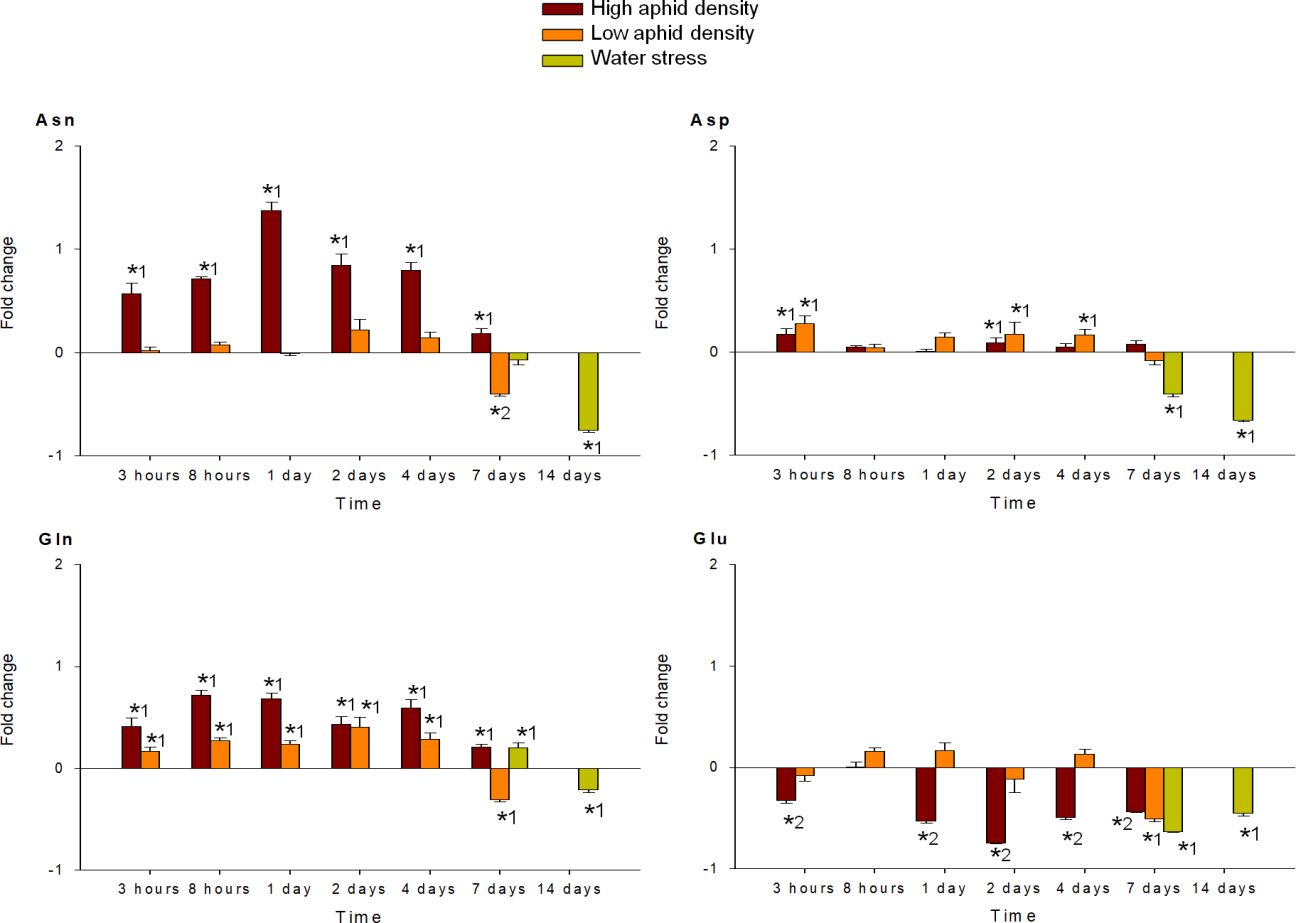
Fold change in Asn, Asp, Gln and Glu of pepper leaves after different treatments (high aphid density, low aphid density, and water stress). Mean +/- s.d. of n = 4. Positive or negative values indicate increases or decreases, respectively. Asterisks indicate significant changes (P-value < 0.05) in Tukey^1^ or Games-Howell^2^ post hoc analysis relative to their respective control leaves.

MANOVA performed on all amino acids analyzed (individual FAA, EAA, NEAA and total FAA) indicated a significant effect of the time of infestation (P < 0.001), for both high and low aphid density treatments. MANOVA performed on the fold-change of each FAA in response to high and low aphid density also revealed a significant effect of the interaction time x density (P < 0.001). Under high aphid density, all amino acids concentrations increased in response to infestation, with exception of Glu, which mostly decreased during the entire study period (Figs 4–9). Most of the 18 amino acids with increased concentrations reached their maximum levels between 1 and 4 dpi. Exceptions to this pattern were Asp, Gln, Hyp, Pro, Ser and Thr, which showed an earlier response with a local maximum before 1 dpi. In quantitative terms, amino acids that more than doubled in concentration in response to high aphid density were, in descending order: Ala, Leu, Lys, Thr, Arg, Tyr, Phe, Val, Ile, Met, Asn and Pro (Figs 4–9). Under low aphid density, most amino acids increased until 2 dpi, followed by a general decrease at 4 dpi that continued until 7 dpi. Amino acids with higher magnitude of variation (increases or decreases greater than 0.3 fold change) were, in descending order: Glu, Asn, Gln, Pro, Ala, Leu, His, Phe, Thr, Tyr and Ser.

### 3.2. Effect of water stress on plant FAA composition. Comparison with aphid infestation

Plants under water constraint showed symptoms of water stress (epinasty and leaf rolling) which were moderate after 7 days but became much more severe after 14 days, when the symptoms were observed in all of the leaves. Water stress resulted in significant changes in the total FAA content (Fig 2), which increased with time. EAA responded earlier than NEAA, with a greater increase after 7 days, although after 14 days the increases in both groups of amino acids were of similar magnitude.

**Fig 10:**
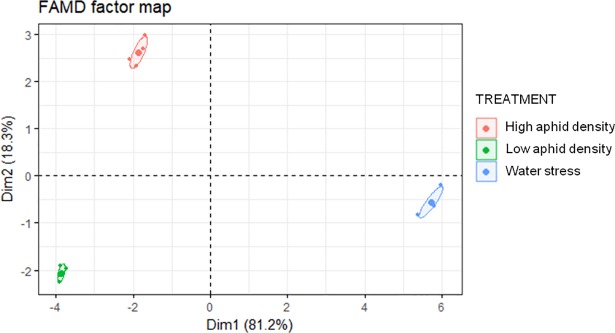
FAMD factor map of FAA in pepper leaves after 7 days of different treatments (high aphid density, low aphid density and water stress).

FAMD of high aphid density, low aphid density and water stress after 7 days of treatment revealed three groups markedly differentiated in their FAA composition (Fig 10). The two first dimensions explained 99.5% of the total variability. Dimension 1 was composed, in order of descending contribution (S3 Table) by Phe Val, “treatment”, Ile, Arg, His, Leu, Trp, Lys, Met, Hyp, Thr, Pro, and Ala. On the other hand, dimension 2 was composed, in order of descending contribution (S3 Table), by the qualitative variable “treatment” and the quantitative variables Asn, Gln, Ser, Asp, and Glu. Variability between aphid infestation (high- and low density) and water stress is mainly explained by the variables belonging to the dimension 1, whereas the variability between high aphid density and low aphid density treatments is mostly explained by the variables belonging to the dimension 2.

MANOVA performed on all amino acids analyzed (individual FAA, EAA, NEAA and total FAA) indicated a significant effect of the time of water stress (P < 0.001). Significance between leaves subjected to 7 or 14 days of water stress, and their corresponding controls in the post hoc analysis, is indicated by asterisks (Figs 4–9). Most amino acids showed an increased concentration in response to water stress, with exception of Asn, Asp, Glu, and Ser, which showed a decrease at 7 days, and also Ala and Glu, which decreased at 14 days. It is worth noting that, although there was an increase in the total FAA content from 7 days to 14 days of water stress (Fig 2), mainly due to the huge increase in Pro and Hyp content (Fig 4), most amino acids became less abundant after 14 days (Figs 5–9). In addition to Pro and Hyp, other amino acids with increased concentrations after 14 days of water stress were Glu, His, Phe, Trp, and Val. Amino acids with greater than two-fold concentration increase in response to water stress, in descending order: Hyp, Pro, Trp, Phe, Ile, His, Leu, Ala, Val, Arg, and Lys.

## 4. Discussion

The two densities of infestation assayed gave very different results in our study. Low aphid density provoked minor variations in the FAA composition of pepper leaves, but there was a significant decrease in total FAA content at the end of the study period (7 dpi). These results are in line with previous studies with different asymptomatic aphids, which showed little or no effect on the FAA content of their host plants [11, 15, 16], especially when compared to symptomatic aphid species causing chlorotic lesions or galls in their plant hosts [7, 10–13]. Thus, it seems that at low aphid density *M*. *persicae* either remain undetected or do not trigger a strong response in pepper plants. Conversely, high aphid density triggered a large increase in total FAA content. Specific amino acids, including the aromatics Phe, Tyr, Trp; the branched-chain amino acids (BCAA), Val, Ile, and Leu; and a miscellaneous group with Arg, Lys, Met, Thr, Ala, Asn, and His, accumulated and peaked at different times post-infestation. This again coincides with previously published works describing strong effects of asymptomatic aphids in plant FAA content, when using aphid densities similar [15] or even higher [14] to our high aphid density. Nevertheless, we have observed a significant interaction of time x density. Therefore, not only the density of infestation is important to define plant amino acid responses, but also the time of exposure.

Generally speaking, most differences between aphid infestation and water stress were quantitative. The most relevant difference was found in the total FAA fold change, which was much higher under water stress than in response to aphid infestation, mainly due to a huge accumulation of Pro and Hyp. Accordingly, previous publications described a large increase in FAA in response to drought and osmotic stress [26, 27, 28]. In addition, water stress induced a higher accumulation of the BCAA group, Phe, Trp, Arg, Lys, Met, His, Thr, and Ala compared to aphid infestation, but a lower accumulation of Ser and the glutamate group (Glu, Gln, Asp, Asn). The existence of shared traits among both stresses is not unexpected given that they involve overlap and interactions between hormone, redox, nitric oxide, kinase, and calcium signaling pathways [29].

In some cases, aphid infestation has been described to specifically increase the content of EAA, which have relatively low abundance in the phloem sap under unstressed conditions [5, 6]. This finding has driven the intriguing hypothesis that aphids may manipulate the composition of phloem sap for their own benefit [11, 12]. However, in our study the increase in total FAA content of pepper leaves was mainly due to a rise in EAA, not only in the case of *M*. *persicae* infestation but also in response to water stress, as also has been described in tomato leaves in response to drought [28]. Although EAA accumulation may be a consequence of an adaptive manipulation by the aphids, the possibility that it is a general plant response to stress must also be considered. EAA for aphids include amino acids that are precursors for a large array of secondary metabolites with defensive or signalling functions in plants [1, 3, 30].

With regard to individual amino acids that respond to plant stress, Pro has been studied the most extensively. Its accumulation primarily occurs in response to stresses that cause dehydration of the plant tissue and it is commonly used as a biochemical marker of water stress (see [31] and references therein). The present results show a drastic increase in Pro and its hydroxylated derivative, Hyp, in pepper leaves in response to water stress, as was previously described by Del Amor *et al*. [27]. Hyp also was shown to accumulate along with Pro in oak leaves in response to water stress [32]. Interestingly, under high aphid density, Hyp levels remained unaltered and Pro content showed a significant decrease at 7dpi. Furthermore, both amino acids peaked at 8 hpi and decreased thereafter. Apart from its role in osmotic adjustment, several functions in stress resistance have been also reported for Pro, including protection of cellular structure during dehydration, redox buffering, storage and transfer of reductants, signaling, and reactive oxygen scavenging (reviewed in [31]). Moreover, Pro content in plants has been negatively correlated with aphid development in the case of *Aphis gossypii* [33] and *M*. *persicae* [34]. Hyp can be used for the synthesis of Hyp-rich glycoproteins, which are also enriched in other amino acids that accumulated in our study, including Ala, Val, Thr, Lys and Tyr [35]. The Hyp-rich glycopeptide systemin has been shown to confer resistance against *Helicoverpa armigera* larvae [36], and Dardeau *et al*. [7] suggested the accumulation of Hyp-rich peptides in aphid-infested tissues. The absence of a substantial accumulation of Pro and Hyp under high aphid density at longer times of infestation (from 2dpi) may be related to a metabolic manipulation of aphids, thus preventing the release of defense signaling pathways. However, this possibility has not been investigated. It is worth mentioning that Ala was the amino acid that increased the most in response to aphid feeding. Although the high fold-change observed for this amino acid may be partly due to its low basal level in unstressed conditions, as for Hyp, we cannot ruled out an active role in the plant response to aphid infestation. Ala may accumulate as a by-product of the γ-aminobutyric acid shunt, which has been associated with various physiological responses, including defense against insects [37].

Amino acids other than Pro that accumulate upon water or osmotic stress include the BCAA group, the aromatics, and Thr, Lys, Arg, and Met [26, 38–41]. Of these, the BCAA showed greater fold changes than the others in response to stress [39]. Their biosynthesis consumes NADPH in the plastids and their catabolism releases reducing agents within the mitochondria, thereby participating in similar redox buffering and energy transfer mechanisms as Pro [31]. However, research with *Arabidopsis* has shown that BCAA accumulate in response to osmotic stress, including water stress, primarily due to protein degradation rather than *de novo* biosynthesis [42]. Other studies have demonstrated accumulation of both BCAA and aromatic amino acids after MeJA treatment [43] and insect feeding [14, 43]. Accordingly, in the present study, BCAA and aromatic amino acids are among the most increased amino acids in response to high aphid density and water stress.

Arg accumulation was also induced by water stress and, to a lesser extent, under high aphid density. Arg was described as a compatible solute in yeast under hyperosmotic stress [44] and was also shown to accumulate in wheat under osmotic stress [26]. Moreover, Arg accumulation may be related to its role as the main precursor of the polyamines putrescine, spermidine, and spermine. Other amino acids that greatly accumulated under stress conditions, especially in the case of high aphid density, were Met and Lys. Met, through its intermediate S-adenosylmethionine, is a precursor for polyamines and ethylene and Lys is the precursor of the diamine cadaverine. Polyamines have been shown to play important roles in plant responses to different abiotic stresses (reviewed in [45]). More recently, the participation of plant amines and their biosynthetic enzymes in the response of plants to aphid infestation has also been described [46].

In contrast to other amino acids, Glu decreased in response to both water stress and aphid infestation. These results are in line with previous publications showing an increase in several amino acids, but not Glu, as a consequence of osmotic stress or phytophagous attack [14, 26, 28]. Glu metabolism participates in numerous plant processes, including nitrogen assimilation, metabolism and transport, carbon/nitrogen partitioning, and stress-associated metabolism. Under stress conditions, Glu metabolism is used for rapid production of stress-associated metabolites [47]. Thus, the decrease in Glu content in our study may be explained by its extensive use for the synthesis of the other strongly induced amino acids; mainly BCAA, aromatic amino acids, and Arg, but also Pro and Hyp in the case of water stress. It is known that, in response to a large variety of abiotic stresses or biotic attacks, plants induce nitrogen remobilization processes in order to translocate and safeguard nutrients in their non-infected tissues [17]. In the present study, Gln and Asn increased after aphid feeding, and Gln also increased under water stress. Diverse studies have pointed out the relevance of these two amino acids in plant defense responses. Gln synthase activity was strongly increased in potato plants after aphid infestation [4], Gln was shown to play a crucial role in *Arabidopsis* disease resistance [48], and Asn synthetase was required for plant nitrogen assimilation and defense against microbial pathogens in pepper plants [49]. Moreover, Gln and Asn are major nitrogen-transport compounds in plants [50]. Interestingly, Gln was the only amino acid showing a significant change at low aphid density throughout the complete period under study. Its accumulation may suggest mobilization of amino acids away from aphids, leading to the significant decrease in total FAA observed at 7 dpi under low aphid density.

In summary, we have shown that the asymptomatic *M*. *persicae* induces significant changes in the FAA composition of pepper leaves, depending on aphid density and time post-infection. These changes were of a lower magnitude than those observed in response to water stress.

## Supporting information

S1 Table Triple quadrupole MRM acquisition method parameters. (PDF)

S2 Table Contribution (%) by dimension of each amino acid in FAMD by time and density of aphid infestation. (PDF)

S3 Table Contribution by dimension (%) of each amino acid in FAMD by treatment. (PDF)
