# Supplementary material for: Changes in the free amino acid composition of Capsicum annuum (pepper) leaves in response to Myzus persicae (green peach aphid) infestation. A comparison with water stress
Source: PLoS One. 2018 Jun 1;13(6):e0198093. doi: 10.1371/journal.pone.0198093 (PMC5983507; doi:10.1371/journal.pone.0198093)
Supplement: S1 Table — (PDF) [file pone.0198093.s001.pdf]

S1 Table. Triple quadrupole MRM acquisition method parameters.

Compound name	Retention time (min)	Precursor ion (m/z)	Product ion (m/z)	Fragmentor (V)	CE (V)
Alanine	0.521	90,09	**44,05**	380	10
			42	380	10
Arginine	1.538	175,2	**70**	380	16
			60,06	380	25
Asparagine	0.424	133,1	87,1	380	4
			**74,1**	380	10
Aspartate	0.442	134,1	88	380	5
			**74**	380	10
Cisteine	0.509	122	76	380	25
			**59**	380	25
Cystine	0.484	241	**151,9**	380	4
			195	380	4
Glycine	0.447	76,07	51,7	380	4
			**29,9**	380	30
Glutamate	0.8	148,1	130,1	380	4
			**84,1**	380	15
Glutamine	0.450	147,1	130,1	380	15
			**84,1**	380	5
Hydroxyproline	0.434	132	**85,9**	380	4
			68	380	4
Histidine	0.822	156,1	**109,9**	380	25
			93,04	380	25
Isoleucine	4.224	132,2	**69,1**	380	4
			56,9	380	20
Leucine	4.289	132,2	**86,1**	380	4
			54,9	380	20
Lysine	0.9	146,9	130,08	380	25
			**83,9**	380	25
Methionine	2.5	150,2	104,05	380	25
			**56,05**	380	25
Phenylalanine	4.5	166,2	**119,9**	380	25
			103,06	380	25
Proline	0.641	116,1	**70**	380	25
			68,05	380	25
Serine	0.436	106,1	88,04	380	15
			**60,05**	380	25
Threonine	0.491	120,1	74,01	380	20
			**56,01**	380	20
Tryptophan	4.843	205,2	**188**	380	4
			146	380	16
Tyrosine	4.089	182	**136**	380	10
			91	380	20
Valine	2.110	118,1	72	380	25
			**55,06**	380	25

Quantifier transitions are marked in bold.
