# Supplementary material for: Changes in the free amino acid composition of Capsicum annuum (pepper) leaves in response to Myzus persicae (green peach aphid) infestation. A comparison with water stress
Source: PLoS One. 2018 Jun 1;13(6):e0198093. doi: 10.1371/journal.pone.0198093 (PMC5983507; doi:10.1371/journal.pone.0198093)
Supplement: S2 Table — (PDF) [file pone.0198093.s002.pdf]

S2 Table. Contribution (%) by dimension of each amino acid in FAMD by time and density of aphid infestation.

	Dim.1	Dim.2	Dim.3	Dim.4	Dim.5
Alanine	6.131	0.400	0.289	0.309	0.031
Arginine	6.195	0.560	0.106	0.07	0.002
Asparagine	5.873	0.767	0.107	0.843	0.465
Aspartate	0.061	4.832	35.390	0.999	1.595
Glutamate	2.178	22.200	0.038	0.544	0.129
Glutamine	4.206	9.252	0.096	0.246	2.574
Hydroxyproline	3.289	3.199	8.876	0.130	7.265
Histidine	4.953	0.139	4.063	2.611	0.348
Isoleucine	6.164	0.696	0.076	0.030	0.077
Leucine	6.142	0.988	0.037	0.010	0.014
Lysine	6.186	0.600	0.008	0.006	0.001
Methionine	5.939	0.692	0.344	0.043	0.123
Phenylalanine	6.215	0.487	0.158	0.001	0.008
Proline	2.889	12.904	2.965	2.135	0.229
Serine	3.387	11.832	0.023	6.011	0.011
Threonine	6.244	0.017	0.275	0.096	0.229
Tryptophan	5.823	0.320	1.146	0.618	0.012
Tyrosine	6.144	0.829	0.138	0.002	0.033
Valine	6.246	0.398	0.098	0.038	0.002
Density	4.707	1.053	2.150	0.012	1.718
Time	1.028	27.874	43.617	85.245	85.133

{Density Levels = High and low aphid densities;
Time levels = 3hpi, 8hpi, 1dpi, 2dpi, 4dpi and 7dpi}
