# Supplementary material for: Changes in the free amino acid composition of Capsicum annuum (pepper) leaves in response to Myzus persicae (green peach aphid) infestation. A comparison with water stress
Source: PLoS One. 2018 Jun 1;13(6):e0198093. doi: 10.1371/journal.pone.0198093 (PMC5983507; doi:10.1371/journal.pone.0198093)
Supplement: S3 Table — (PDF) [file pone.0198093.s003.pdf]

S3 Table. Contribution (%) by dimension of each amino acid In FAMD by treatment.

	Dim.1	Dim.2	Dim.3	Dim.4	Dim.5
Alanine	4.906	4.109	0.444	1.141	28.944
Arginine	5.848	0.0625	0.045	0.178	3.120
Asparagine	0.455	23.923	0.213	1.147	1.172
Aspartate	4.243	6.741	19.228	2.278	0.226
Glutamate	4.180	6.598	68.296	4.035	1.918
Glutamine	2.530	14.561	5.542	9.173	13.346
Hydroxyproline	5.676	0.843	0.272	0.096	0.344
Histidine	5.821	0.173	0.378	1.354	0.324
Isoleucine	5.855	0.007	0.239	1.470	4.949
Leucine	5.811	0.245	0.010	0.354	0.051
Lysine	5.711	0.592	3.764	1.202	3.852
Methionine	5.690	0.699	0.015	1.947	4.911
Phenylalanine	5.863	0.005	0.318	0.144	2.403
Proline	5.634	0.971	0.168	1.334	9.059
Serine	2.897	13.041	0.151	5.476	10.593
Threonine	5.664	0.756	0.075	10.061	5.890
Tryptophan	5.801	0.288	0.022	0.00002	0.754
Tyrosine	5.692	7.152	0.247	2.091	0.507
Valine	5.863	5.332	0.002	1.363	0.916
Treatment	5.859	2.567	0.570	3.410	6.721

{Treatment levels = High aphid density, low aphid density, and water constraint}
